# Cell Cycle Arrest and Cell Survival Induce Reverse Trends of Cardiolipin Remodeling

**DOI:** 10.1371/journal.pone.0113680

**Published:** 2014-11-25

**Authors:** Yu-Jen Chao, Wan-Hsin Chang, Hsiu-Chi Ting, Wei-Ting Chao, Yuan-Hao Howard Hsu

**Affiliations:** 1 Department of Chemistry, Tunghai University, Taichung, Taiwan; 2 Department of Life Science, Tunghai University, Taichung, Taiwan; 3 Life Science Research Center, Tunghai University, Taichung, Taiwan; Universidad Pablo de Olavide, Centro Andaluz de Biología del Desarrollo-CSIC, Spain

## Abstract

Cell survival from the arrested state can be a cause of the cancer recurrence. Transition from the arrest state to the growth state is highly regulated by mitochondrial activity, which is related to the lipid compositions of the mitochondrial membrane. Cardiolipin is a critical phospholipid for the mitochondrial integrity and functions. We examined the changes of cardiolipin species by LC-MS in the transition between cell cycle arrest and cell reviving in HT1080 fibrosarcoma cells. We have identified 41 cardiolipin species by MS/MS and semi-quantitated them to analyze the detailed changes of cardiolipin species. The mass spectra of cardiolipin with the same carbon number form an envelope, and the C64, C66, C68, C70 C72 and C74 envelopes in HT1080 cells show a normal distribution in the full scan mass spectrum. The cardiolipin quantity in a cell decreases while entering the cell cycle arrest, but maintains at a similar level through cell survival. While cells awakening from the arrested state and preparing itself for replication, the groups with short acyl chains, such as C64, C66 and C68 show a decrease of cardiolipin percentage, but the groups with long acyl chains, such as C70 and C72 display an increase of cardiolipin percentage. Interestingly, the trends of the cardiolipin species changes during the arresting state are completely opposite to cell growing state. Our results indicate that the cardiolipin species shift from the short chain to long chain cardiolipin during the transition from cell cycle arrest to cell progression.

## Introduction

DNA damage, nutrition deprivation and oxidative stress can lead to cell cycle arrest, and the irreversible damages trigger apoptosis or necrosis of the cells [1], [2]. The repaired cells exit the arrested state and reenter cell cycle, which are highly regulated by cyclin and cyclin-dependent kinase complexes [3]–[5]. Besides protein and DNA synthesis, phospholipids are synthesized and turn over in G1, double the mass in S, and pause the synthesis in G2 and M for cell division [6]. The mitochondrial phospholipid cardiolipin also accumulated during HeLa cell entry into the S phase [7]. Phospholipases and transacylases are important enzymes to incorporate and switch fatty acyl chains of phospholipid for maturation [8]–[10]. It has been shown that phosphocholine cytidylyltransferase activity increases in G1 and decreases steadily through late S and G2/M, which reversely coordinates with net phospholipid accumulation on membrane [11]. Hydrolysis of phospholipid by phospholipases produces lysophospholipids and the fatty acid derived eicosanoids, which have been shown to relate to various cellular signaling [9].

During cell survival and cell progression, mitochondria in particular have mitochondrial genome to regulate their own replication [12], [13]. Cardiolipin is a major membrane component maintaining the integrity of mitochondria [14]–[16] and critical for ATP production via the electron transport chain [17]–[19]. The electron transport chain complexes are stabilized by binding to cardiolipin on the inner mitochondrial membrane [20], [21]. Oxidative stress can cause the oxidation of cardiolipin and thus, interferes with the electron transport chain and alters the mitochondrial bioenergetics [22]–[24]. The mitochondrial alterations in cancer cells, Barth Syndrome patients and diabetic mouse models have shown to be accompanied with mitochondrial cardiolipin changes [25]–[27]. Cardiolipin binds to cytochrome *c* to regulate the release of cytochrome *c*, which prevents mitochondria rupture in an early apoptosis event [28]–[30]. However, apoptosis can also be triggered by the activation of peroxidase activity of cardiolipin-bound cytochrome *c* [31].

Cardiolipin uniquely has one big anionic head group with two negative charges from the phosphates and four fatty acyl chains. This negatively charged head group and the hydrophobic tails commonly interact with protein complexes, such as electron transport chain complex II, III and IV and ATP synthase, on the mitochondrial membrane through electrostatic attractions and hydrophobic interactions to stabilize their structures [20], [21], [32]–[36]. The four fatty acyl chains are commonly found symmetrical in normal cells, such as C18:2 in mammalian heart and C22:6 and C20:5 in mollusk bivalves [37], [38]. The cells lost the regular function of cardiolipin synthesis and remodeling to maintain the symmetry of cardiolipin in mouse brain tumors [39]. The commercially available cardiolipin from *E. coli* extract (Avanti Lipids) is asymmetrical containing unique cyclo-C17:0 in the main species. More cardiolipin species have been found in methane-metabolizing archaea [40]. It has been found that phospholipase A_2_s can hydrolyze cardiolipin to produce monolysocardiolipin, dilysocardiolipin and fatty acids [41].

Cardiolipin is a critical phospholipid to maintain the function and the morphology of mitochondria [13], [42]–[44]. In the mitochondrial cell cycle, cardiolipin is most likely going through the sequence of synthesis, remodeling, and replication process before mitochondrial mitosis [45]. We have established HT-1080 fibrosarcoma as a cell line for cardiolipin analysis. In this research, we examined the quantity and species changes of all cardiolipin by mass spectrometry and discovered how cell cycle arrest and survival affect the species and quantity of cardiolipin.

## Materials and Methods

### Materials

HT-1080 human fibrosarcoma cell line and MCF-7 human breast cancer cell line were kindly provided by Dr. Wei-Ting Chao. Dulbecco's modified eagle medium (DMEM), fetal bovine serum (FBS), penicillin/streptomycin and 1M HEPES for cell culture were purchased from Gibco, USA. Tetramyristoyl cardiolipin standard (14:0)_4_ and tetralinoleoyl cardiolipin (18∶2)_4_ extracted from bovine heart were purchased from Avanti Polar Lipids, USA.

### Cell Starvation (Synchronization) and Survival

HT-1080 and MCF-7 cells were cultured in Dulbecco's modified eagle medium with 10% fetal bovine serum, 0.5% penicillin-streptomycin and 25 mM HEPES in 5% CO_2_ at 37°C. The cells were grown to 50% confluent in 10-cm culture dish for cell survival group and 6-cm culture dish for starvation group, removed the medium and washed with PBS. The cells were then cultured in serum-free medium for 48 hr. During the time course of serum starvation, the cells were harvested at 0, 12, 24, 36 and 48 hr. After serum starvation, the cells were activated by adding growth medium back to cells, and the cells were collected at 0, 4, 8, 16 and 22 hr. All experiments were done in triplicates.

### Flow Cytometry

The harvested cells (1–2×10^6^ cells) were rinsed with PBS buffer and placed in the 15 ml centrifuge tube. The cells were carefully resuspended, added pre-cooled 70% ethanol and stored in 4°C overnight. The fixed cells were rinsed by PBS buffer twice, treated with 2 µg of Rnase A and stained with propidium iodide (50 µg/ml in PBS) at 37°C for 30 min. The prepared cells were subjected to flow cytometer (Moxi flow, ORFLO) without gating.

### Lipid Extraction

Total lipid in collected cells were extracted according to the Bligh-Dyer's method [46]. 125 ng of (14:0)_4_ cardiolipin was added as an internal standard to the cell pallets in 2ml methanol. After sonication, 1 mL of chloroform was added into samples to maintain the ratio of chloroform/methanol  = 1∶2, and vortexed for 10 min. Then, 1 ml of chloroform and 1 ml of distilled deionized water were added to samples and further vortexed for 15 min. The lower phase in the glass test tube was collected after centrifugation at 2000 rpm for 5 min.

### Mass Spectrometry Analysis

The extracted total lipid was dried under nitrogen gas and re-dissolved in 200 µl of acetonitrile/2-propanol/H_2_O (65∶30∶5). 20 µl of the samples were analyzed by LC/MS Ion-Trap (Bruker). HPLC mobile phases were solution A: ACN∶H_2_O (60∶40), 10 mM ammonium formate, 0.1% formic acid and solution B: IPA∶ACN (90∶10), 10 mM ammonium formate, 0.1% formic acid [47]. Gradient was from 60% solution A to 100% solution B in 25 min and maintained 100% solution B until 45 min in an Acclaim RSLC 120 C18 2.1 mm ×100 mm 2.2 µm column (Thermo) at a flow rate of 0.2 mL/min at 55°C. Data were further analyzed by Bruker DataAnalysis (ver.3.4). The extract ion current (EIC) of each cardiolipin species was quantitated by their relativity of EIC to internal standard. The total cardiolipin is the sum of all quantitated cardiolipin species. Standard deviations are calculated for the error bars of the histograms and t-tests are applied to all triplicated data.

## Results

### Cardiolipin Identification

**Figure 1:**
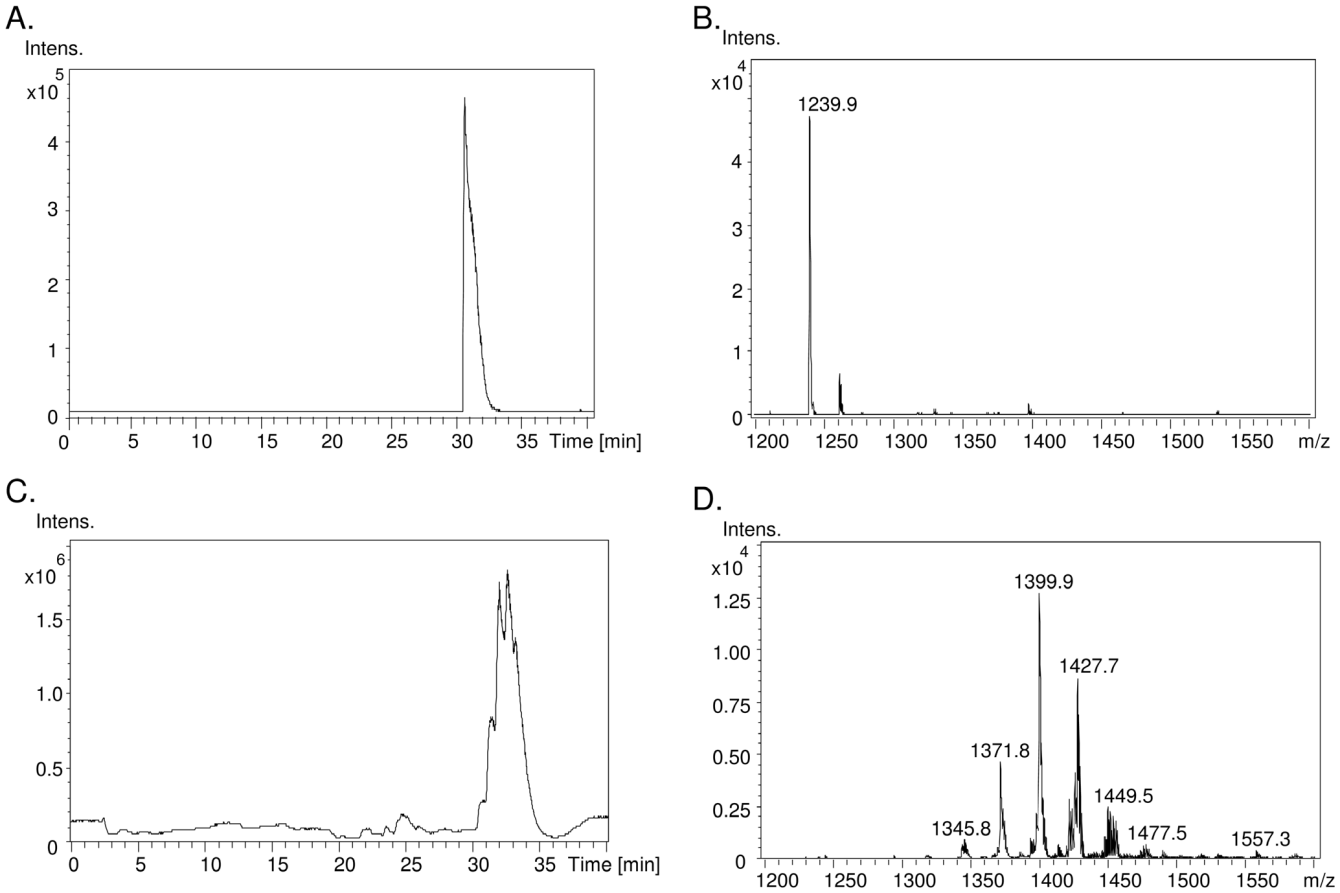
Mass spectrum of cardiolipin in HT1080 cells. A. Total ion chromatogram and B. mass spectrum of cardiolipin standard (14:0)_4_ are shown. C. Total ion chromatogram and D. mass spectrum of cardiolipin extracted from HT1080 cells are shown.

**Figure 2:**
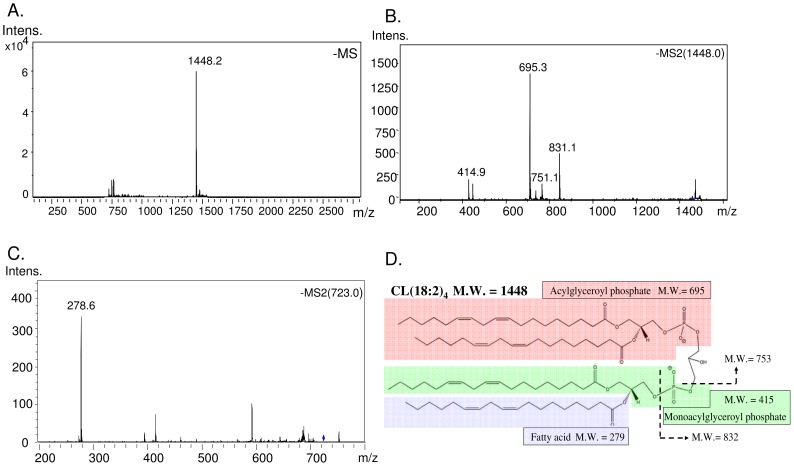
Identification of the cardiolipin species by tandem mass spectrometry. A. A major species of tetralinoleoyl cardiolipin (C18:2) in bovine heart. B. Tandem mass spectrometry identified fragments of tetralinoleoyl cardiolipin. C. Fragmentation of cardiolipin releases fatty acyl chain. D. Fragmentation of cardiolipin releases large fragments including acyl chains.

Based on the unique structure and electrostatic potential of cardiolipin, reverse phase liquid chromatography was applied to separate cardiolipin from other lipid molecules, which were extracted along with cardiolipin by Bligh/Dyer's method. The cardiolipin species extracted from the HT1080 cells are eluted at 30–36 min, except some minor species eluted 2–3 min earlier (Fig. 1). The externally added tetramyristoyl cardiolipin standard m/z 1239.9 is eluted at 31 min at the front of the HT-1080 cardiolipin. Because mass spectrometer detects cardiolipin in negative ion mode, the positively charged phosphatidylcholine (PC) and weak anionic lipid phosphatidylethanolamine (PE) can be excluded in the acidic condition. Cardiolipin also has higher molecular weight of 1200–1600 Da than PG, PS, PI and PA with the mass below 900 Da. All cardiolipin species form a normal distribution of 6 groups by their carbon number in the fatty acyl chain. Each group contains several cardiolipins with various double bonds. Although different species of cardiolipin can be isolated and detected in LC-MS, the molecular weight alone is not enough to identify their four fatty acyl chains. To further confirm the cardiolipin structure, product ion mode of tandem mass spectrometry was applied to analyze every cardiolipin species. A commonly seen tetralinoleoyl-cardiolipin m/z 1448.2 is shown as an example of fragmentation on the Ion-Trap analyzer (Fig. 2A, B). The product ions m/z 831, 751, 695 and 415 are identified to contain either one or two fatty acyl chains (Fig. 2D). Alternatively, fragmentation on the tetralinoleoyl cardiolipin with net charge of -2 generates a different product ion pattern, containing a dissociated linoleic acid m/z 278.6. (Fig. 2C, D). We identify the accurate structure of cardiolipin species based on the above methods.

### Cardiolipin Species in HT1080

To examine the cardiolipin changes during cell survival, HT1080 cell line was utilized as a cell model to examine the cardiolipin species. Total lipids in the harvested cells were extracted by Bligh-Dyer's extraction method and analyzed by LC-MS. A total of 41 cardiolipin species from 31 mass peaks passes the screening threshold, including the synthesized standard (Table 1). The number of carbon in the four acyl chains and the double bonds represents the 31 mass peaks, such as C70:4. The chain lengths of the fatty acids in cardiolipin are in the range of 14–22 carbon, but mainly with 16, 18 and 20 carbons. Worth to note that, each mass peak contains 1–3 species of cardiolipin.

**Table 1:** Cardiolipin species identification in HT-1080 cells.

Species	m/z	Formula
C56:0	1239.7	**(14:0)(14:0)(14:0)(14:0)**
C64:4	1343.3	(16:1)(14:0)(16:1)(18:2)*
		**(18:2)(14:0)(18:2)(14:0)**
C64:3	1345.5	**(16:1)(14:0)(16:1)(18:1)**
C66:5	1369.7	**(16:1)(18:2)(18:2)(14:0)**
C66:4	1371.7	**(18:2)(18:1)(16:1)(14:0)**
C66:3	1373.5	**(18:1)(16:1)(16:1)(16:0)**
C68:8	1391.5	*(34:5)* a **(16:1)(18:2)**
C68:6	1395.7	**(18:2)(14:0)(18:2)(18:2)**
		**(18:2)(18:2)(16:1)(16:1)**
		(16:1)(18:2)(16:1)(18:2)*
C68:5	1397.7	**(16:1)(16:1)(18:1)(18:2)**
		(16:1)(18:1)(16:1)18:2)*
C68:4	1399.3	**(16:1)(16:0)(18:1)(18:2)**
		(16:1)(18:1)(16:1)(18:1)*
C68:3	1401.3	(16:1)(16:1)(18:1)(18:0)*
		**(16:1)(16:0)(18:1)(18:1)**
		(16:1)(18:0)(16:1)(18:1)*
C70:6	1423.5	**(16:1)(18:1)(18:2)(18:2)**
C70:5	1426.1	**(16:1)(18:1)(18:1)(18:2)**
C70:4	1427.8	**(16:1)(18:1)(18:1)(18:1)**
C72:9	1446.4	**(18:2)(18:2)(18:2)(18:3)**
		*(34:4)* a **(20:4)(18:1)**
C72:7	1450.7	**(18:2)(18:2)(18:2)(18:1)**
C72:6	1451.8	**(18:2)(18:2)(18:1)(18:1)**
C72:5	1453.5	**(18:2)(18:1)(18:1)(18:1)**
C72:4	1455.8	**(18:1)(18:1)(18:1)(18:1)**
C74:10	1472.5	**(16:0)(18:2)(20:4)(20:4)**
C74:9	1474.4	**(18:2)(18:2)(18:2)(20:3)**
C74:7	1477.8	**(18:1)(18:2)(18:2)(20:2)**
C76:13	1493.4	**(16:1)(18:2)(20:4)(22:6)**
C76:12	1495.5	**(18:2)(18:2)18:2)(22:6)**
C76:11	1497.7	(18:1)(18:2)(18:2)(22:6)*
		**(18:2)(18:2)(20:3)(20:4)**
C76:10	1499.5	**(18:2)(18:1)(18:2)(22:5)**
C78:13	1521.4	*(36:4)* a **(20:3)(22:6)**
C78:12	1523.5	**(18:2)(18:2)(20:4)(22:4)**
C78:11	1525.4	**(18:1)(18:1)(20:3)(22:6)**
		(18:1)(18:2)(20:2)(22:6)*
C78:9	1529.7	**(16:1)(20:4)(20:4)(22:0)**

**Bold:** identified species;

*: main species;

a *a*: partially identified formula.

### Cardiolipin Quantity and Species in Cell Survival

**Figure 3:**
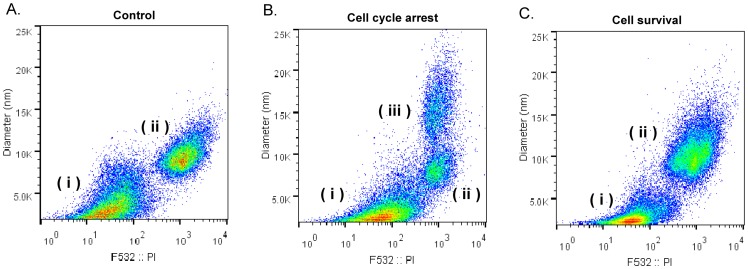
Flow cytometry analysis of the cell states after serum starvation. After HT-1080 cells undergo serum deprivation for 48 hr, the cells revive by replenishing serum for 22 hr. A. Regular growing cells, B. cell cycle arrested cells and C. cell survival cells were harvested, fixed and treated with propidium iodide for DNA staining, and then analyzed on flow cytometry. Three arbitrary groups of cells on the map were labeled as i, ii and iii.

To examine the changes of cardiolipin species in cell survival, the cells underwent serum starvation for 48 hours to arrest the cells in G0 phase. The cells stopped growing after serum deprivation, but no significant cell death or growth was observed under microscope and the total protein measurement (data not shown). Replenishing the serum activated the cells to escape from G0/G1 phase, and the reviving cells were harvested at 0, 4, 8, 16 and 22 hr. Flow cytometry detects the transitions of DNA replication and cell dimensions in the cell phases during cell cycle arrest and cell survival (Fig. 3). Growing cells have 57% in G1 phase (i) and 39.4% in S/G2 phase (ii) with duplicated DNA (Fig. 3A). After serum starvation for 2 days, three groups of the cells indicate 68.4% of cells are arrested in G0/G1 (i), 14.0% of cells are arrested in S/G2 phase (ii) and 13.2% are arrested in G2/M phase (iii). Replenishing serum revive cells back to the similar phase distribution as the regular growing cells.

**Figure 4:**
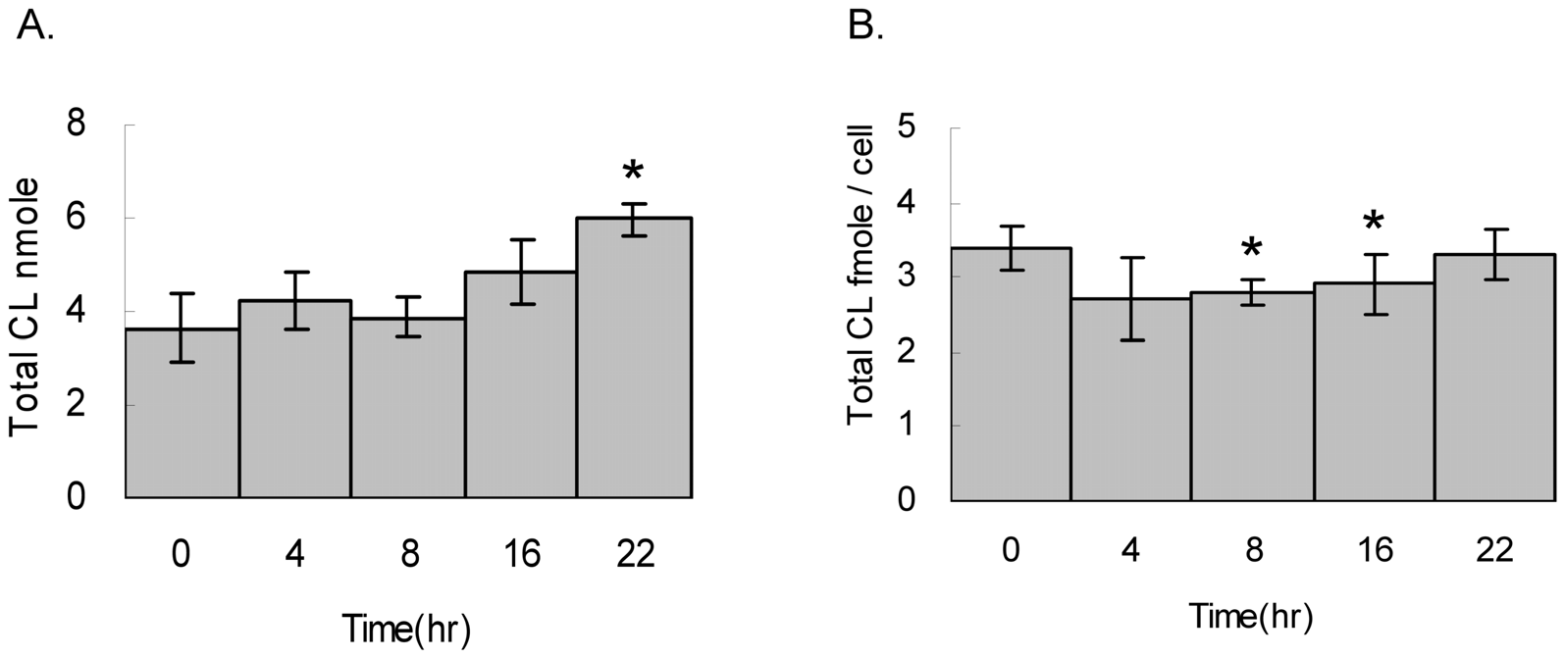
Total cardiolipin quantity after exit of cell cycle arrest. Cells underwent serum starvation for 48 hr, replenished with serum and harvested at 0, 4, 8, 16 and 22 hr in triplicates. A. The total lipids are extracted and cardiolipin are quantitated by LC-MS. B. The total cardiolipin per cell are in triplicate and calculated by t-tests.

Cardiolipin species in the total lipid extract of the HT1080 cells were measured by LC-MS. The cell number and the total cardiolipin both show similar trend of increase after 16 and 22 hours of cell survival (Fig. 4). The results also indicate the increase of the cardiolipin coordinated with cell replication and the quantity of cardiolipin per cell does not have a sudden increase at any specific time point.

**Figure 5:**
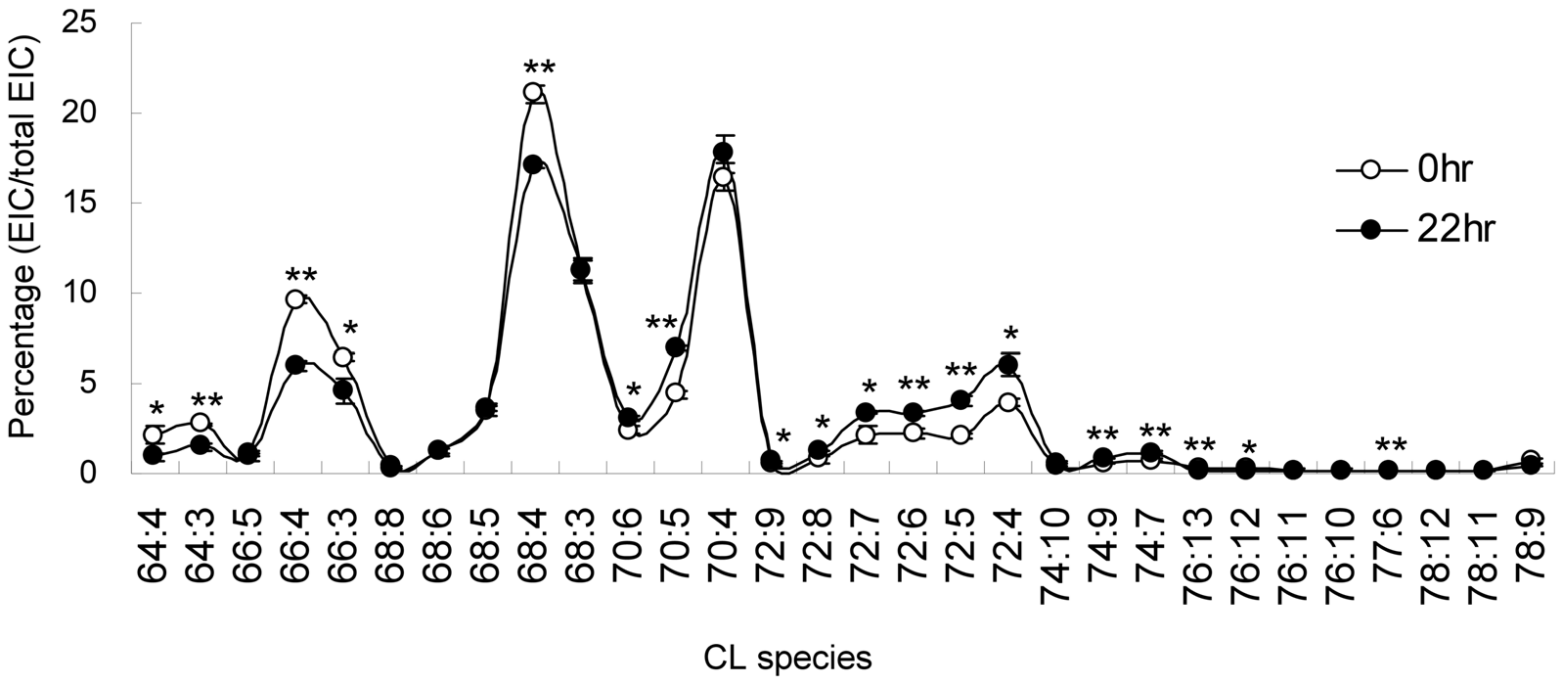
Shifting of Cardiolipin Species in Cell Cycle. HT1080 cells were arrested by serum starvation, and revived from 10% FBS. Cells were harvested at 22 hr for cardiolipin extraction in triplicates. Cardiolipin species were measured by mass spectrometry and normalized by total extracted ion counts among cardiolipin species. T-tests are applied to all triplicated samples.

Each species of cardiolipin is semi-quantitated by the relative extracted ion current to tetramyristoyl cardiolipin. Although the total cardiolipin per cell kept at a similar level through the 22-hr time course, the species and quantity of cardiolipin in a cell may be affected during cell survival and the arrested state. Our results show that the cardiolipin species changes are not dramatic, but the percentage of cardiolipin species shows a steady variation through cell survival (Fig. 5).

### Shift of the Cardiolipin Species

**Figure 6:**
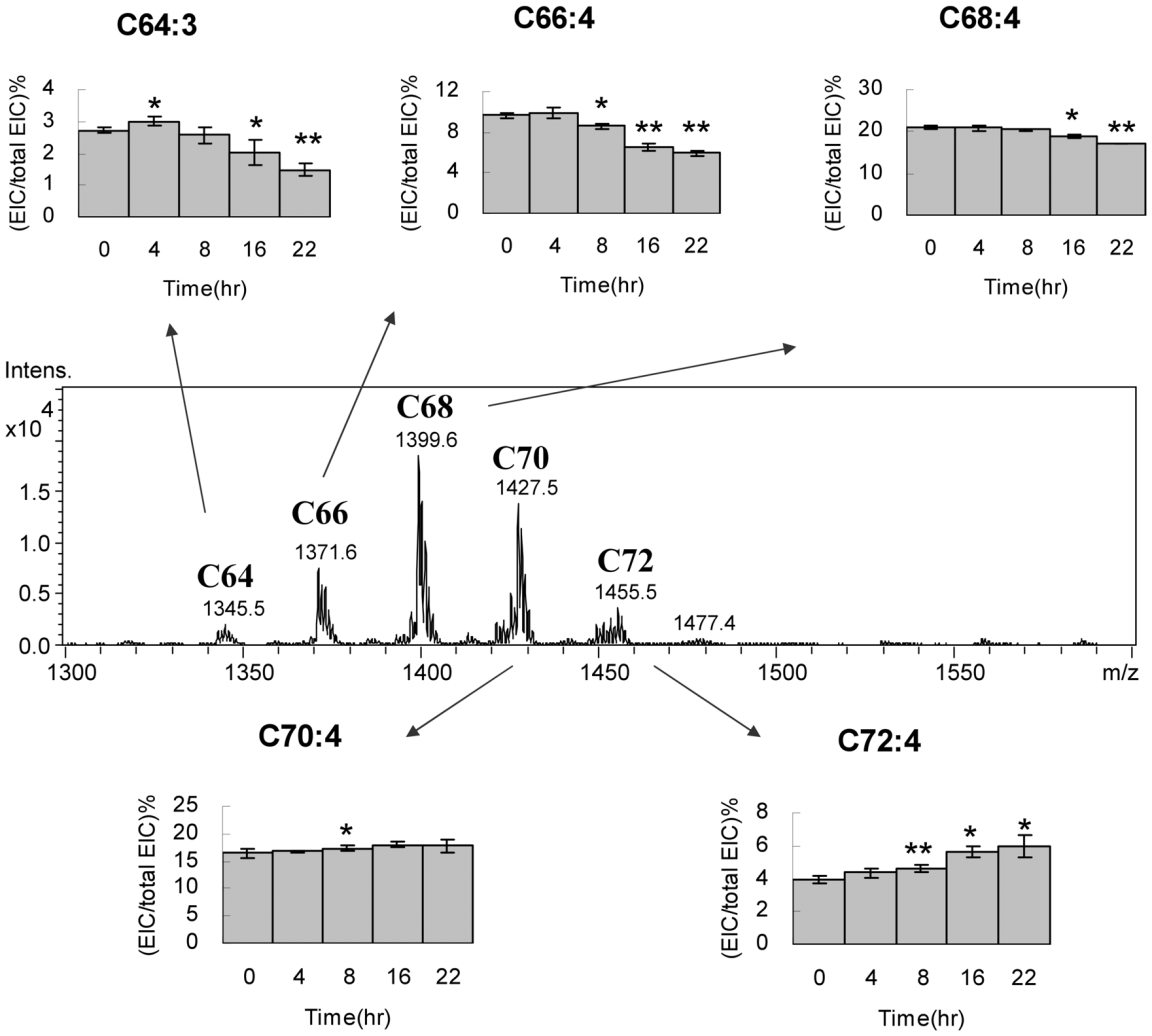
Cardiolipin content shifting in carbon groups. The cardiolipin groups are labeled above the mass peak. The species with the highest peak intensity in the groups are selected to show the percentage changes at the 22-hr time course. T-tests are applied to all triplicated samples.

The C64, C66, C68, C70 C72 and C74 groups of Cardiolipin in HT1080 cells display a normal distribution on the mass spectrometry spectrum (Fig. 6). Addition of every two carbons to cardiolipin structure increases 28 Da to the molecular weight of cardiolipin. The C68 group shows the strongest intensity. The quantity of cardiolipin declines along with the increase or decrease of carbon number. Among the 6 cardiolipin groups, the C66, C68, C70 and C72 combine for 70% of total cardiolipin content, and C64, C74 are two minor groups. One double bond in the fatty acyl chain reduces the cardiolipin mass by 2 Da. As a result, the cardiolipins with the same number of carbon but different number of double bond form a mass envelope. The changes of cardiolipin percentages of each species within the same group carry similar trends. Therefore, the species with the highest quantity in each mass envelope presents the group change in cell survival. Although the trends in each species may still slightly vary, the C64, C66 and C68 groups with low carbons show decreasing cardiolipin percentage, whereas the C70 and C72 groups with high carbons show increasing cardiolipin percentage.

**Figure 7:**
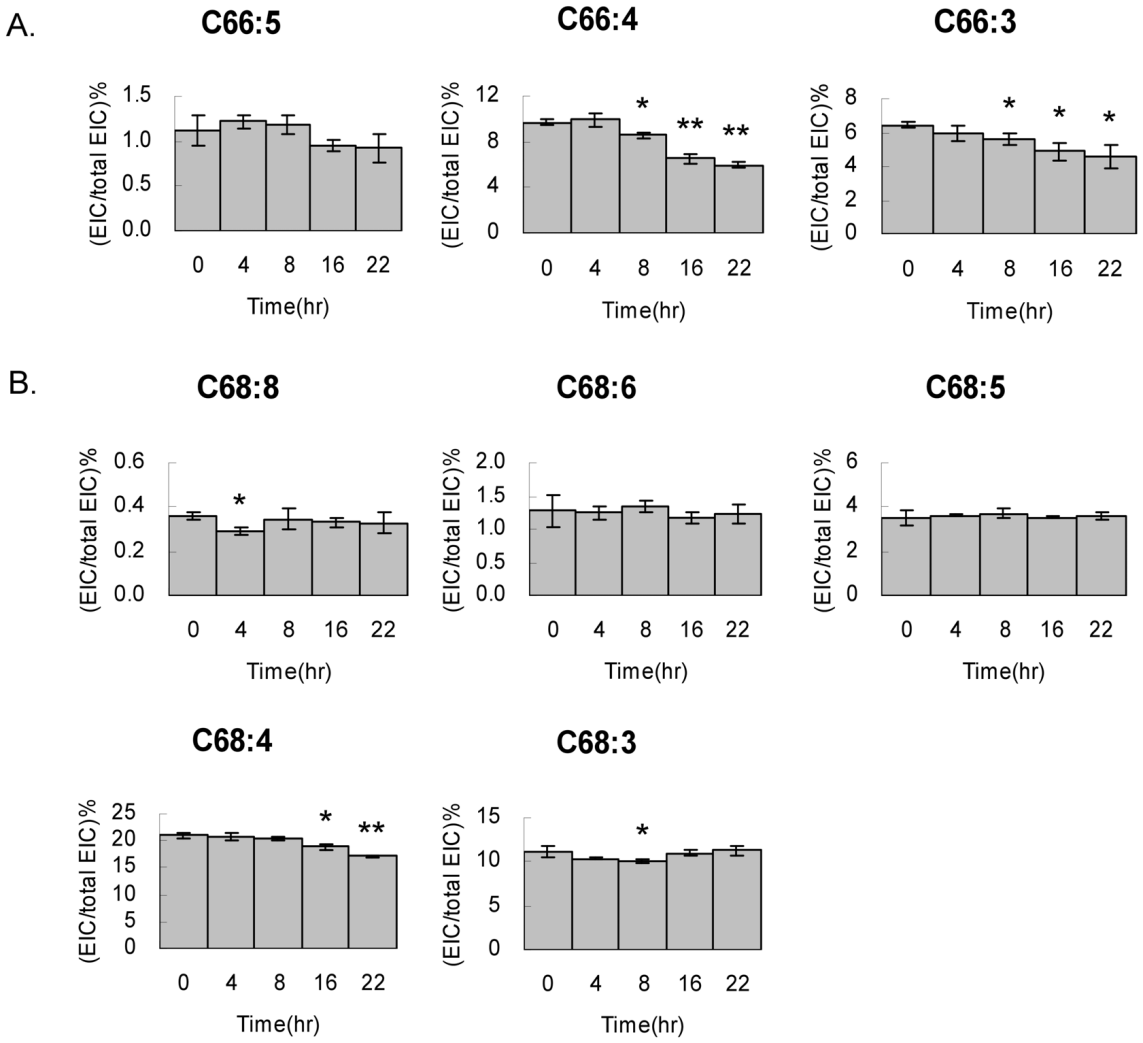
Cardiolipin content decrease in C66 and C68 groups. HT1080 cells exited cell cycle arrest and harvested at 0, 4, 8, 16 and 22 hr for cardiolipin extraction in triplicates and then subjected for LC-MS analysis. All cardiolipin species in A. C66 and B. C68 are quantitated by extracted ion current relative to the (C14:0)_4_ cardiolipin standard. The cardiolipin percentage are calculated and plotted against the survival time. T-tests are applied to all triplicated samples.

In the C66 group, C66:3, C66:4 and C66:5 show decreases of cardiolipin contents after cell survival (Fig. 7A). Among them, C66:4 and C66:3 accounts for 9.6% and 6.4% of the total cardiolipin in the cell respectively. After 22 hr, the contents drop 3.6% and 1.9% of the total cardiolipin, which are 37% and 18% decrease relative to the C66:4 and C66:3 controls at the cell arrest state. Similar to the trend of C66, the C64:3 cardiolipin decreases 45% relative to the C64:3 control after 22 hours of cell reviving. The C68 group does not show significant changes in the full time course, including C68:3, C68:4, C68:5, C68:6 and C68:8 (Fig. 7B). Each species shows similar quantity except C68:4, the most abundant cardiolipin in the C68 group and in the HT1080 cell, showing a unique 19% decrease relative to the C68:4 control. The trends of decreases in these cardiolipin species were further confirmed in another independent experiment in triplicates (Fig. S1 in File S1).

**Figure 8:**
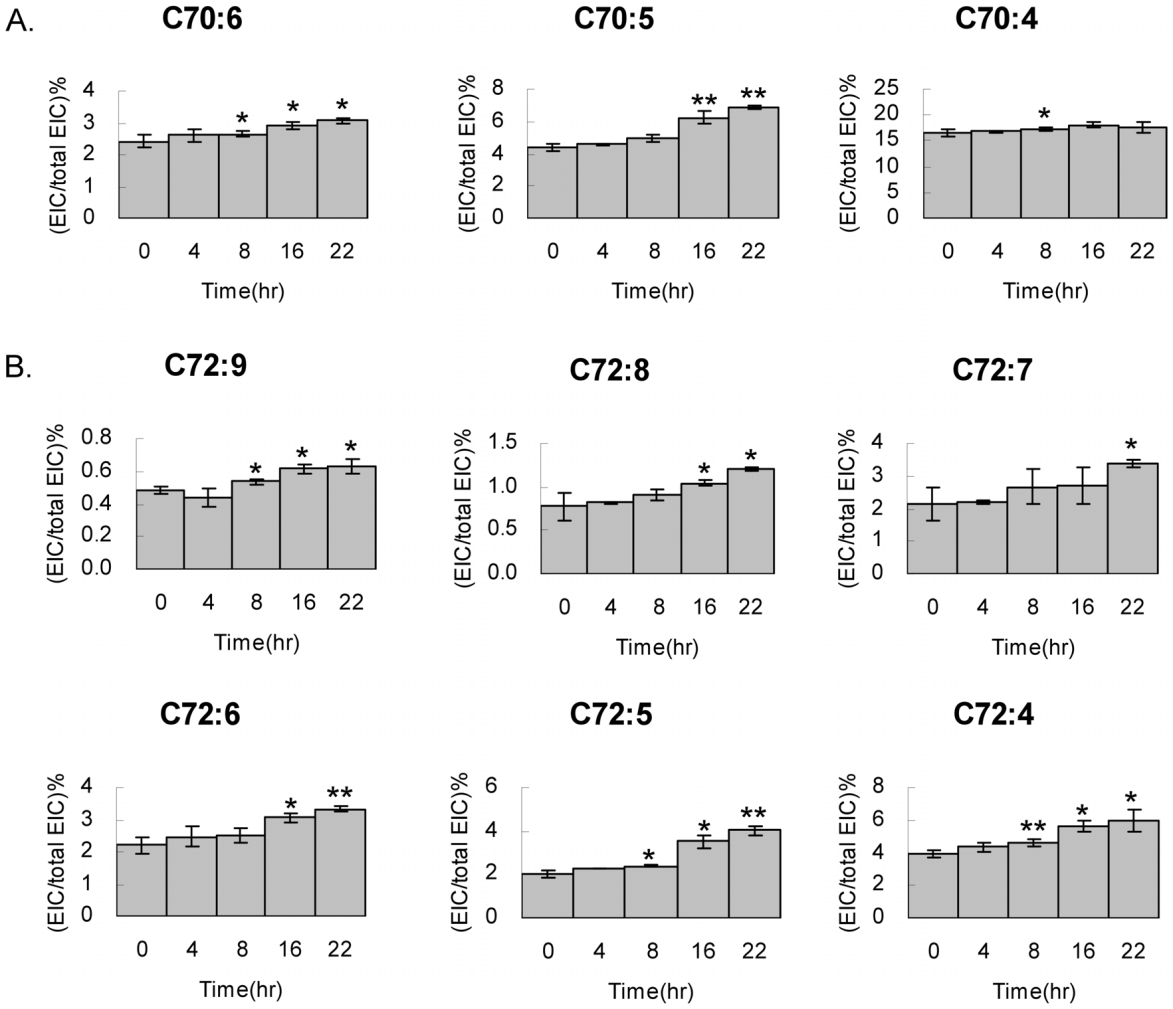
Cardiolipin content increased in C70 and C72 groups. HT1080 cells exited cell cycle arrest and harvested at 0, 4, 8, 16 and 22 hr for cardiolipin extraction in triplicates and then subjected for LC-MS analysis. All cardiolipin species in A. C70 and B. C72 are quantitated by extracted ion current relative to the (C14:0)_4_ cardiolipin standard. The cardiolipin percentages are in triplicate, calculated by t-tests and plots against the survival time.

For the cardiolipin with carbon number above 70, their cardiolipin contents increase through cell survival. In the C70 group, C70:4, C70:5 and C70:6 show 1.3%, 2.5% and 0.7% increase of the total cardiolipin, accounting for 10%, 36% and 23% increases of the relative species controls at the arrested state (Fig. 8). The C72:4 and C72:5 cardiolipin also show significant 33% and 50% increases of the C72:4 and C72:5 controls after cell arrest exit. The trends of decreases in these cardiolipin species were also further confirmed (Fig. S2 in File S1).

### Cardiolipin Species Shifting during Cell Arrest

**Figure 9:**
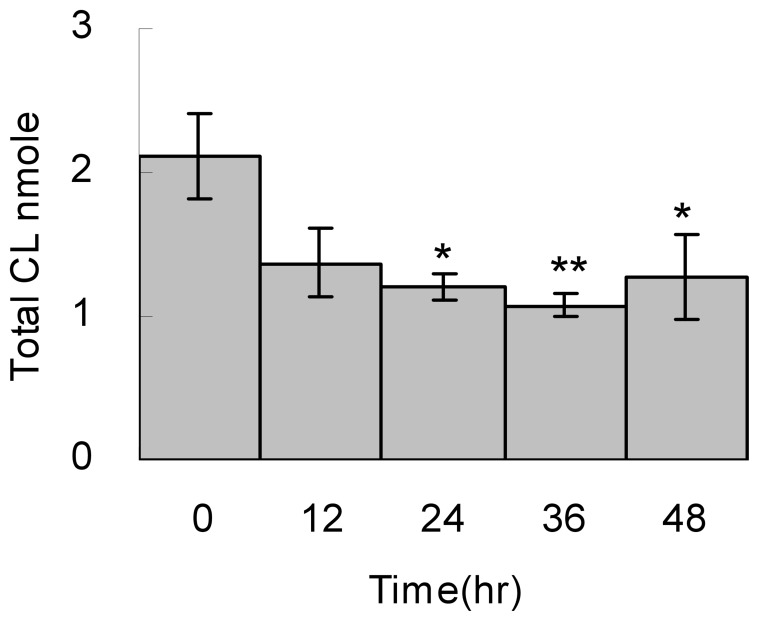
Total cardiolipin quantity while entering cell cycle arrest. Cells underwent serum starvation for 0, 12, 24, 36 and 48 hr and then harvested for total lipid extraction in triplicates. The cardiolipin quantity was analyzed by LC-MS. The extract ion current (EIC) of each cardiolipin species was quantitated and the total cardiolipin is the sum of all cardiolipin species. T-tests are applied to all triplicated samples.

Cell cycle arrest is a cellular state for energy conservation or damage repair, which affects the mitochondrial activity and the ATP production. We hypothesize that the cardiolipin effects we have seen above during cell survival shall have reverse effects during cell cycle arrest. Therefore, we applied serum starvation for 2 days to examine the cardiolipin changes. Indeed, the total cardiolipin quantity has an average of 35% decrease after 12 hours to 2 days of serum starvation, which is a reverse effect observed in the cell survival experiment (Fig. 9). Because cell number does not increase after serum starvation, this result indicates the cells lost their cardiolipin while entering cell cycle arrest.

**Figure 10:**
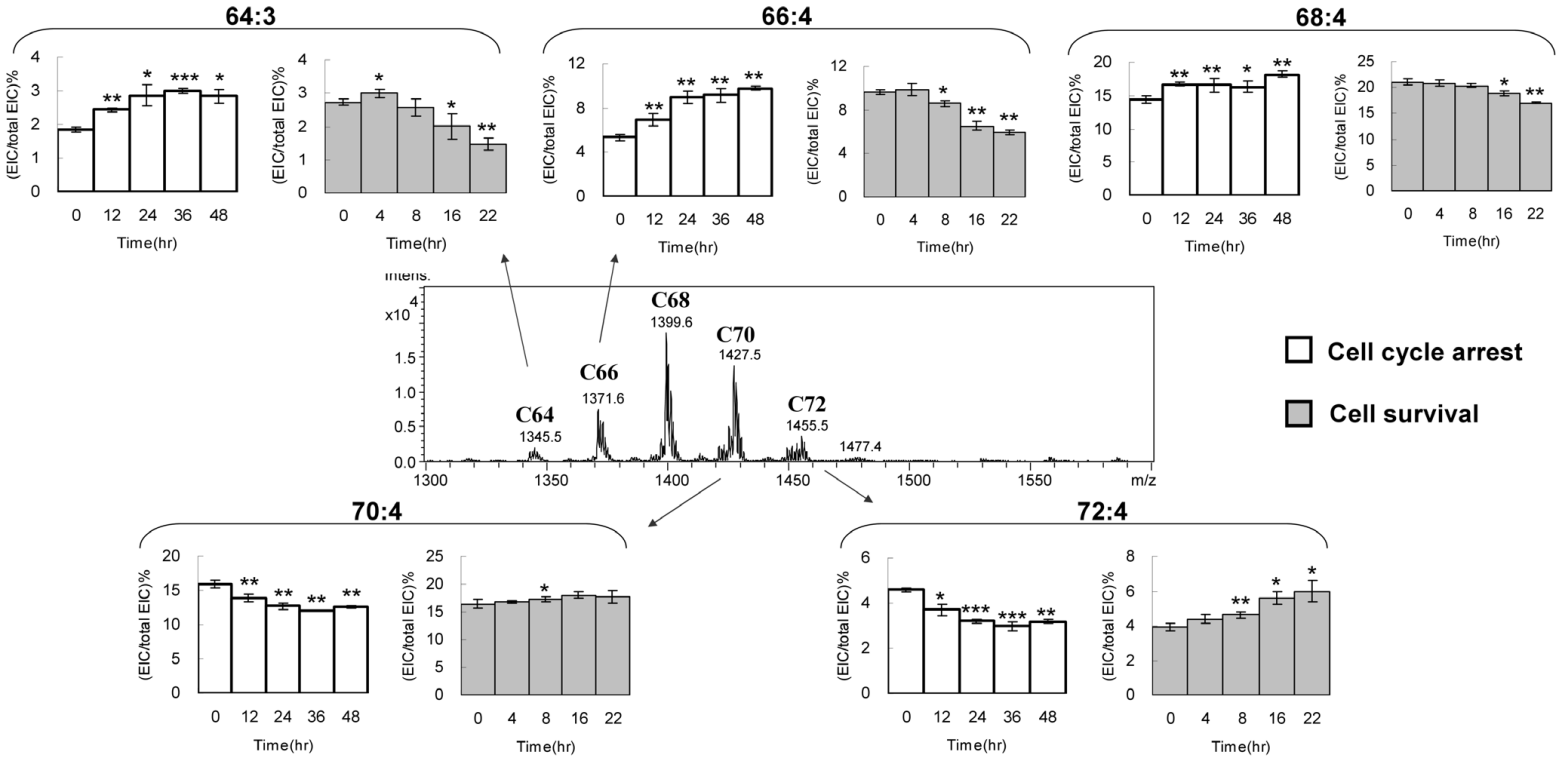
Comparisons of cardiolipin species shift between cell arrest and cell survival in HT1080. The cardiolipin groups are labeled above the mass peak. The species with the highest peak intensity in the groups are selected to show the percentage changes at the 22-hr time course. T-tests are applied to all triplicated samples.

To understand whether the species of cardiolipin changes along with the decrease of the total quantity, the cardiolipin species are further analyzed (Fig. S3 in File S1). The overall species after starvation are the similar to that after cell survival. However, the trends between cell cycle arrest and cell survival are completely opposite (Fig. 10). We select the representative cardiolipin species with the highest intensities in each group for comparisons. In the C64 and C66 groups, the cardiolipin percentages substantially increase within 24 hr. The cardiolipin percentage in the C68 groups shows minor increases. On the contrary, the cardiolipin percentage in the C70 and C72 groups significantly decrease within 24 hr. All of the changes between 24 to 48 hr are following the same trends with minor variations. The rest of the cardiolipin species is following the same trend of increase (Fig. S4 in File S1) or decrease (Fig. S5 in File S1) in the whole 48-hr time course.

**Figure 11:**
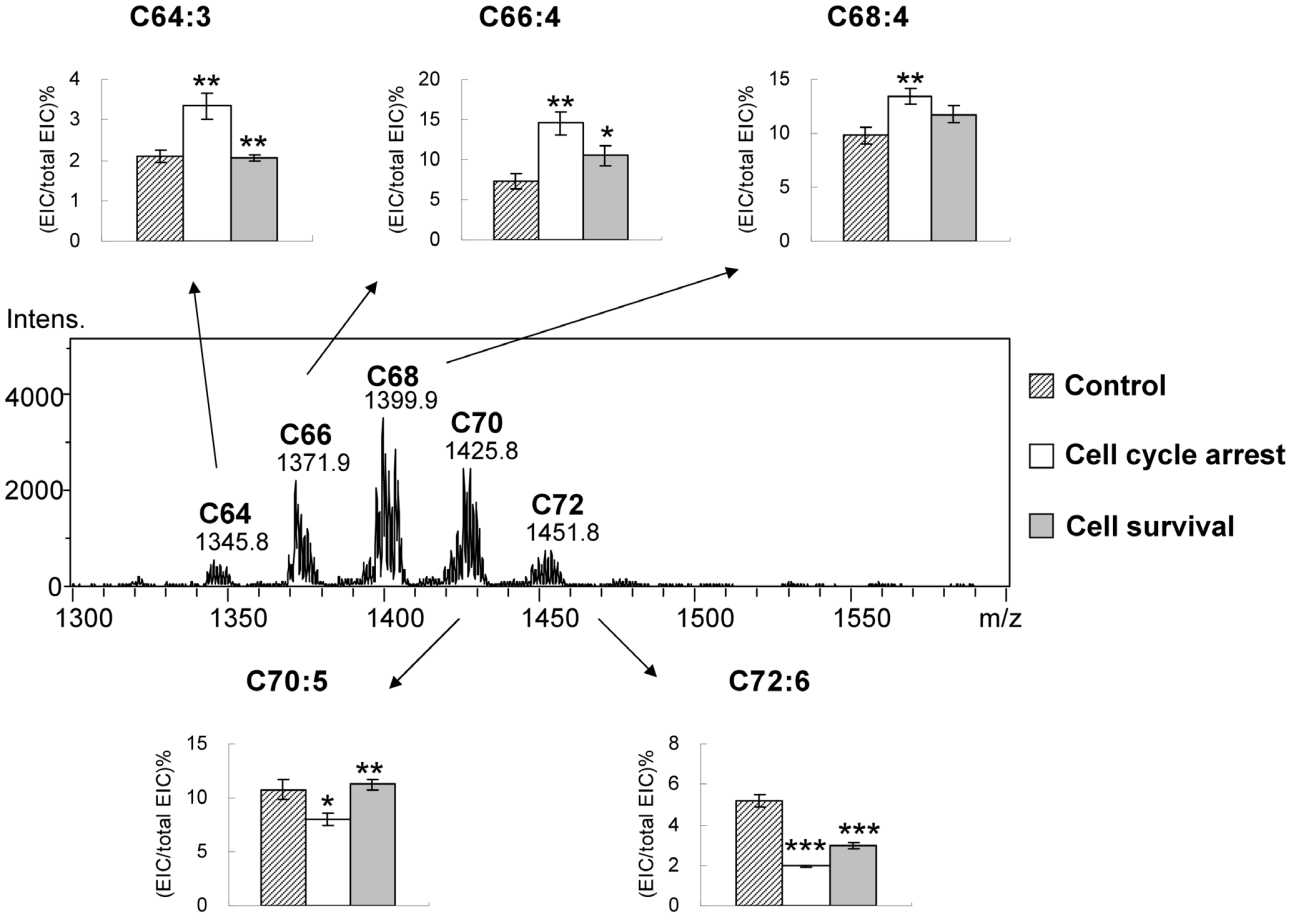
Confirmation of cardiolipin species shift in MCF7. The cardiolipin groups are labeled above the mass peak. The species with the highest peak intensity in the groups are selected to show the percentage changes at the 22-hr time point. The cardiolipin of the regular growing cells, the arrested cells and the reviving cells are quantitated and shown in histograms. T-tests are applied to all triplicated samples.

To further confirm the changes of the cardiolipin profile, we tested the serum deprivation and replenishing effects on the MCF-7 breast cancer cells (Fig. 11). As we expected, MCF-7 cells have the same responses to the serum deprivation and stimulation as the HT-1080 cells, which indicates this effect is not specific to HT-1080.

## Discussion

Cell cycle arrest can lead to cancer dormancy, which can be an important method for cancer therapy, but also a potential problem for further treatment [48]. Awakening of the dormant cancer cell results in cancer recurrence and threaten the patients undergoing chemotherapy. It has been reported that the cancer cells undergo lipid composition changes in mitochondria, which decreases the susceptibility to stimuli and the efficiency of ATP production [49], [50]. Mitochondrial cardiolipin has been found to be symmetrical and mainly C18:2 in human, however, a number of oxidative cardiolipin species have been discovered to cause the asymmetry [51]. Cancer cells lost the symmetry caused by the inability to remodel the cardiolipin, leading to the accumulation of the immature cardiolipin [52]. Indeed, the cardiolipin species in the fibrosarcoma cell line HT1080 have a different pattern as the mammalian tissues, such as pig heart, pig liver and bovine heart, which contains mainly the tetralinoleoyl-cardiolipin, tested previously in our laboratory. We have found that cancer cells will not revert to the symmetrical cardiolipin in different phases in the cell cycle. The cell arrested state, the cell progressing state, the overgrown state and even the cell death state of HT1080 all displayed a normal distribution of the groups of the cardiolipin species. Although the total quantity of cardiolipin increases, the total cardiolipin per cell is not changed after awakening from cell cycle arrest. This implies that the synthesis and breakdown of cardiolipin have maintained balanced from cell cycle arrest state through the cell replication, which is different from our initial hypothesis that cardiolipin might have obvious changes in quantity. However, while cells were entering the cell cycle arrest and maintaining alive, the cardiolipin quantity substantially decreased, indicating a less requirement of cardiolipin and its function.

There are more than 40 asymmetrical cardiolipin species identified in the HT1080 cells, which is a phenomenon caused by lacking of remodeling ability seen in cancer cells. It has been reported that the turnover of cardiolipin is slower than other phospholipids [53]. The species changes observed in our experiments are continuous within 1 day for both cell cycle arrest and cell survival. The versatile cardiolipin species are shifting from the high carbon number to low carbon number cardiolipin in cell cycle arrest; and vice versa in cell survival. From the changes of the cardiolipin species, we found that the fatty acyl chains with 18 and 20 carbons are significantly reduced during serum starvation. Carbon number of cardiolipin is very likely to be a key factor to regulate mitochondrial function after the cells enter or exit cell cycle. The double bonds do not seem to be the main factor to affect cardiolipin remodeling, although the high carbon cardiolipin tends to have high number of double bonds.

Under conditions of low energy, heterotrimeric AMPK is activated in high AMP to alter both consumption and production of ATP [54]. Recently, activated AMPK was shown to phosphorylate Ser15 of p53 to protect the protein from degradation and promote cell-cycle arrest during DNA damage and aberrant growth factor signaling [55]. Upon deletion of p53, nutrient deprivation results in continued cellular proliferation, with an eventual loss in cell viability, confirming the essential requirement for p53 in nutrient-dependent cell-cycle arrest [55]. Our results suggest that the HT1080 cancer cell line does not require highly efficient mitochondria to make ATP in the dormancy. Therefore, mitochondrial cardiolipin was substituted with short carbon chain and transport the long carbon fatty acyl chain for cellular maintenance. The carbon maybe used for bio-energy production or bio-molecule synthesis. During cell survival state, cell cycle arrest in G0G1 to the escape towards the S phase has triggered the shift of the low carbon chain to a higher carbon chain. The cardiolipin behavior during cell cycle arrest and revival taken at the level of acyl chain length may modulate the capacity of cardiolipin to induce mitochondrial membrane curvature. This also indicates the cardiolipin remodeling enzymes are active to switch the fatty acyl chain. The mechanism to avoid the cardiolipin to recover to the long chain cardiolipin may be a method to prevent cancer cell proliferation.

## Supporting Information

File S1 **Supporting figures.** Figure S1, Cardiolipin content increased in C70 and C72 groups. HT1080 cells exited cell cycle arrest and harvested every 2 hours for 22 hr for cardiolipin extraction in triplicates and then subjected for LC-MS analysis. All cardiolipin species in A. C70 and B. C72 were quantitated by extracted ion current relative to the (C14:0)_4_ cardiolipin standard. The cardiolipin percentage were calculated and plotted against the survival time. (value  =  mean ± sd, n = 3). Figure S2, Cardiolipin content decrease in C64, C66 and C68 groups. HT1080 cells exited cell cycle arrest and harvested every 2 hours for 22 hr for cardiolipin extraction in triplicates and then subjected for LC-MS analysis. All cardiolipin species in A. C64 and C66, and B. C68 were quantitated by extracted ion current relative to the (C14:0)_4_ cardiolipin standard. The cardiolipin percentage were calculated and plotted against the survival time. (value  =  mean ± sd, n = 3). Figure S3, Shifting of Cardiolipin Species after serum starvation. HT1080 cells were cultured under serum starvation for 48 hr. The cells were harvested at 0, 12, 24, 36 and 48 hr for cardiolipin extraction in triplicates. Cardiolipin species were measured by mass spectrometry and normalized by total extracted ion counts among cardiolipin species. (value  =  mean ± sd, n = 3). Figure S4, Cardiolipin content increase in C66 and C68 groups after serum starvation. HT1080 cells were cultured under serum starvation for 48 hr. The cells were harvested at 0, 12, 24, 36 and 48 hr for cardiolipin extraction in triplicates and then subjected for LC-MS analysis. All cardiolipin species in A. C66 and B. C68 were quantitated by extracted ion current relative to the (C14:0)_4_ cardiolipin standard. The cardiolipin percentage were calculated and plotted against the survival time. (value  =  mean ± sd, n = 3). Figure S5, Cardiolipin content decrease in C70 and C72 groups after serum starvation. HT1080 cells were cultured under serum starvation for 48 hr. The cells were harvested at 0, 12, 24, 36 and 48 hr for cardiolipin extraction in triplicates and then subjected for LC-MS analysis. All cardiolipin species in A. C70 and B. C72 were quantitated by extracted ion current relative to the (C14:0)_4_ cardiolipin standard. The cardiolipin percentage were calculated and plotted against the survival time. (value  =  mean ± sd, n = 3).
(DOCX)
