# Supplementary material for: Cell Cycle Arrest and Cell Survival Induce Reverse Trends of Cardiolipin Remodeling
Source: PLoS One. 2014 Nov 25;9(11):e113680. doi: 10.1371/journal.pone.0113680 (PMC4244155; doi:10.1371/journal.pone.0113680)
Supplement: File S1 — Supporting figures. Figure S1, Cardiolipin content increased in C70 and C72 groups. HT1080 cells exited cell cycle arrest and harvested every 2 hours for 22 hr for cardiolipin extraction in triplicates and then subjected for LC-MS analysis. All cardiolipin species in A. C70 and B. C72 were quantitated by extracted ion current relative to the (C14:0)4 cardiolipin standard. The cardiolipin percentage were calculated and plotted against the survival time. (value = mean ± sd, n = 3). Figure S2, Cardiolipin content decrease in C64, C66 and C68 groups. HT1080 cells exited cell cycle arrest and harvested every 2 hours for 22 hr for cardiolipin extraction in triplicates and then subjected for LC-MS analysis. All cardiolipin species in A. C64 and C66, and B. C68 were quantitated by extracted ion current relative to the (C14:0)4 cardiolipin standard. The cardiolipin percentage were calculated and plotted against the survival time. (value = mean ± sd, n = 3). Figure S3, Shifting of Cardiolipin Species after serum starvation. HT1080 cells were cultured under serum starvation for 48 hr. The cells were harvested at 0, 12, 24, 36 and 48 hr for cardiolipin extraction in triplicates. Cardiolipin species were measured by mass spectrometry and normalized by total extracted ion counts among cardiolipin species. (value = mean ± sd, n = 3). Figure S4, Cardiolipin content increase in C66 and C68 groups after serum starvation. HT1080 cells were cultured under serum starvation for 48 hr. The cells were harvested at 0, 12, 24, 36 and 48 hr for cardiolipin extraction in triplicates and then subjected for LC-MS analysis. All cardiolipin species in A. C66 and B. C68 were quantitated by extracted ion current relative to the (C14:0)4 cardiolipin standard. The cardiolipin percentage were calculated and plotted against the survival time. (value = mean ± sd, n = 3). Figure S5, Cardiolipin content decrease in C70 and C72 groups after serum starvation. HT1080 cells were cultured under serum starvation [file pone.0113680.s001.docx]

Figure S1: Cardiolipin content increased in C70 and C72 groups. HT1080 cells exited cell cycle arrest and harvested every 2 hours for 22 hr for cardiolipin extraction in triplicates and then subjected for LC-MS analysis. All cardiolipin species in A. C70 and B. C72 were quantitated by extracted ion current relative to the (C14:0)_4_ cardiolipin standard. The cardiolipin percentage were calculated and plotted against the survival time. (value = mean ± sd, n=3)

Figure S2: Cardiolipin content decrease in C64, C66 and C68 groups. HT1080 cells exited cell cycle arrest and harvested every 2 hours for 22 hr for cardiolipin extraction in triplicates and then subjected for LC-MS analysis. All cardiolipin species in A. C64 and C66, and B. C68 were quantitated by extracted ion current relative to the (C14:0)_4_ cardiolipin standard. The cardiolipin percentage were calculated and plotted against the survival time. (value = mean ± sd, n=3)

Figure S3: Shifting of Cardiolipin Species after serum starvation. HT1080 cells were cultured under serum starvation for 48 hr. The cells were harvested at 0, 12, 24, 36 and 48 hr for cardiolipin extraction in triplicates. Cardiolipin species were measured by mass spectrometry and normalized by total extracted ion counts among cardiolipin species. (value = mean ± sd, n=3)

Figure S4: Cardiolipin content increase in C66 and C68 groups after serum starvation. HT1080 cells were cultured under serum starvation for 48 hr. The cells were harvested at 0, 12, 24, 36 and 48 hr for cardiolipin extraction in triplicates and then subjected for LC-MS analysis. All cardiolipin species in A. C66 and B. C68 were quantitated by extracted ion current relative to the (C14:0)_4_ cardiolipin standard. The cardiolipin percentage were calculated and plotted against the survival time. (value = mean ± sd, n=3)

Figure S5: Cardiolipin content decrease in C70 and C72 groups after serum starvation. HT1080 cells were cultured under serum starvation for 48 hr. The cells were harvested at 0, 12, 24, 36 and 48 hr for cardiolipin extraction in triplicates and then subjected for LC-MS analysis. All cardiolipin species in A. C70 and B. C72 were quantitated by extracted ion current relative to the (C14:0)_4_ cardiolipin standard. The cardiolipin percentage were calculated and plotted against the survival time. (value = mean ± sd, n=3)
